# Correlation between amino acid residues converted by RNA editing and functional residues in protein three-dimensional structures in plant organelles

**DOI:** 10.1186/1471-2229-8-79

**Published:** 2008-07-16

**Authors:** Kei Yura, Mitiko Go

**Affiliations:** 1 Graduate School of Humanities and Sciences, Ochanomizu University, 2-1-1, Otsuka, Bunkyo, Tokyo 112-8610, Japan; 2 Ochanomizu University, 2-1-1, Otsuka, Bunkyo, Tokyo 112-8610, Japan; 3 Department of Bio-Science, Faculty of Bio-Science, Nagahama Institute of Bio-Science and Technology, 1266, Tamura-cho, Nagahama, Shiga 526-0829, Japan

## Abstract

**Background:**

In plant organelles, specific messenger RNAs (mRNAs) are subjected to conversion editing, a process that often converts the first or second nucleotide of a codon and hence the encoded amino acid. No systematic patterns in converted sites were found on mRNAs, and the converted sites rarely encoded residues located at the active sites of proteins. The role and origin of RNA editing in plant organelles remain to be elucidated.

**Results:**

Here we study the relationship between amino acid residues encoded by edited codons and the structural characteristics of these residues within proteins, e.g., in protein-protein interfaces, elements of secondary structure, or protein structural cores. We find that the residues encoded by edited codons are significantly biased toward involvement in helices and protein structural cores. RNA editing can convert codons for hydrophilic to hydrophobic amino acids. Hence, only the edited form of an mRNA can be translated into a polypeptide with helix-preferring and core-forming residues at the appropriate positions, which is often required for a protein to form a functional three-dimensional (3D) structure.

**Conclusion:**

We have performed a novel analysis of the location of residues affected by RNA editing in proteins in plant organelles. This study documents that RNA editing sites are often found in positions important for 3D structure formation. Without RNA editing, protein folding will not occur properly, thus affecting gene expression. We suggest that RNA editing may have conferring evolutionary advantage by acting as a mechanism to reduce susceptibility to DNA damage by allowing the increase in GC content in DNA while maintaining RNA codons essential to encode residues required for protein folding and activity.

## Background

RNA editing is a process that inserts, deletes and converts nucleotides in RNA after transcription, distinct from RNA splicing and 3' processing [1,2]. The insertion/deletion type of RNA editing was first discovered in protozoan kinetoplastid mRNAs [3], and the conversion type of RNA editing was first discovered in the mammalian mRNA encoding apolipoprotein B (*apoB*) [4,5], followed by its discovery in the plant mitochondrial mRNA for *coxII *[6] and chloroplast mRNA for *rpl2 *[7]. Since then, conversion editing has been mostly found in mRNAs transcribed from the mitochondrial and chloroplast genomes of land plants [8-12]. In the mitochondrion of *Arabidopsis thaliana*, at least 441 nucleotides in mRNAs are subjected to RNA editing [13]. In the chloroplast of black pine, at least 26 sites are edited [14]; in the chloroplast of the hornwort *Anthoceros formosae*, 942 RNA editing sites have been identified [11]. The majority of conversion editing events in plant organelles occur within protein-coding regions of mRNAs, and involve cytidine-to-uridine (C-U) and sometimes uridine-to-cytidine (U-C) conversion [2]. Earlier analysis [15] of the location of RNA targets within transcripts did not detect any rules to explain why particular residues within a protein sequence were affected by codon changes while other residues were not altered.

RNA editing often increases the percent identity of the encoded amino acid sequence to the homologous sequences, implying an important role for RNA editing in the function of encoded proteins. In maize chloroplast *rpl2*, the AUG initiation codon is generated by conversion of ACG [16]. In cytochrome *c *oxidase subunit II, encoded by the mitochondrial DNA of *Zea mays*, a codon for a copper ligand residue was converted from the codon for Thr to that for Met; Met is required at the site to bind a copper ion, which is in turn prerequisite for electron transfer, the biological function of cytochrome *c *oxidase [17]. In wheat mitochondrion ORF240, equivalent to cytochrome *c *biosynthesis protein, RNA editing converts a codon for one of the heme-binding residues to encode an amino acid appropriate for the heme interaction [18]. Unedited *psbF *mRNA of spinach chloroplast causes a photosystem II-deficient phenotype [19]; unedited *petB *mRNA of tobacco chloroplast causes a defect in heme attachment to cytochrome *b*_6 _[20]; and unedited acetyl-coA carboxylase carboxyl transferase β of pea is not functional [21]. These examples are exceptional cases that demonstrate the functional importance of RNA editing; for the majority of RNA editing events in organelles, however, the functional importance has not been specifically elucidated.

Genome sequencing and structural genomics projects have produced massive amounts of data, including RNA editing sites, organelle genome sequences, and protein 3D structures. In this report, we combine these data and computationally investigate implications for the functional roles of RNA editing. We define protein functions through protein 3D structures, and find that residues converted by RNA editing have significant bias toward structurally important sites.

## Results

### Conversion-type RNA editing in DNA sequence databases

In Genbank release 158, there are 365 genes in plant organelles that undergo conversion editing at 3,560 nucleotides within their protein-coding regions (Table 1). Among these, 1,219 RNA editing events are observed in the first nucleotide of a codon, 1,983 events in the second nucleotide, and 358 events in the third nucleotide. The number of edited nucleotides in a codon is not limited to one. Out of the 3,560 events, 129 pairs of RNA editing events are targeted to the same codon within a gene. The first and second nucleotides of a codon are edited in 86 cases, the second and third in 33 cases, and the first and third in 10 cases.

**Table 1:** Products of mRNAs undergoing conversion editing

**Protein Name**	**# of genes**	**# of edited nucleotide sites**	**PDB ID (chain)**
**Transcription machinery**			

RNA polymease α	2	24 (2:21:1)	2a69 (A), 1coo (_)
RNA polymerase β	1	19 (6:13:0)	2a69(C)
RNA polymerase β'	2	20 (9:11:0)	2a69 (D)

**Translation machinery**			

ribosomal protein S1	2	6 (1: 5: 0)	
ribosomal protein S2	4	34 (12: 22: 0)	2j00 (B)
ribosomal protein S3	7	74 (20: 32: 22)	2j00 (C)
ribosomal protein S4	4	52 (18:30:4)	2j00 (D)
ribosomal protein S7	4	9 (2:7:0)	
ribosomal protein S8	1	2 (1: 1: 0)	2j00 (H)
ribosomal protein S10	2	5 (2: 3: 0)	2j00 (J)
ribosomal protein S12	3	21 (4: 14: 3)	2j00 (L)
ribosomal protein S13	3	18 (5: 12: 1)	2j00 (M)
ribosomal protein S14	3	5 (1:3:1)	2j00(N)
ribosomal protein S19	5	33 (7:19:7)	2j00(S)
ribosomal protein L2	4	10 (3:4:3)	2j01(D)
ribosomal protein L5	5	28 (8:20:0)	2j01(G)
ribosomal protein L14	1	1 (1:0:0)	2j01(O)
ribosomal protein L16	4	33 (6:19:8)	2j01(Q)
ribosomal protein L20	1	3 (1:2:0)	2j01(U)
ribosomal protein L21	1	2 (2:0:0)	2j01(V)
ribosomal protein L22	1	2 (0:2:0)	2j01(W)
ribosomal protein L23	1	3 (0:3:0)	2j01(X)
ribosomal protein L33	1	1 (0:1:0)	2j01(6)
ribosomal protein L36	1	1 (0:1:0)	
translation initiation factor 1	1	3 (1:2:0)	1hr0(W)
Clp protease proteolytic subunit 1	1	6 (0:6:0)	1yg6(A)

**Respiratory machinery**			

NADH dehydrogenase subunit J	1	2 (0:2:0)	2fug(5)
NADH dehydrogenase subunit K	1	3 (1:1:1)	2fug(6)
NADH dehydrogenase subunit I	1	2 (1:1:0)	2fug(9)
NADH dehydrogenase subunit 1	23	141 (71:60:10)	
NADH dehydrogenase subunit 2	25	236 (55:147:34)	
NADH dehydrogenase subunit 3	7	102 (36:62:4)	
NADH dehydrogenase subunit 4	10	111 (31:77:3)	
NADH dehydrogenase subunit 4L	9	64 (15:48:1)	
NADH dehydrogenase subunit 5	7	180 (44:94:42)	
NADH dehydrogenase subunit 6	5	61 (14:40:7)	
NADH dehydrogenase subunit 7	4	86 (20:49:17)	
NADH dehydrogenase subunit 9	6	58 (24:33:1)	
cytochrome *bc*1 complex cytochrome *b*	7	136 (68:59:9)	1kyo(C)
cytochrome *c *biogenesis ccmC	5	156 (65:77:14)	
cytochrome *c *biogenesis ccmF	15	195 (90:88:17)	
cytochrome *c *biogenesis ccsA	1	12 (2:10:0)	
cytochrome c ccmB	7	278 (108:145:25)	
cytochrome *c *oxidase I	20	324 (107:196:21)	1v55(A)
cytochrome *c *oxidase II	14	164 (64:85:15)	1v55(B)
cytochrome *c *oxidase III	15	155 (42:82:31)	1v55(C)

**Photosynthesis machinery**			

photochlorophyllide reductase subunit chlL	1	9 (5:4:0)	2afh(E)
photochlorophyllide reductase subunit chlB	8	23 (10:13:0)	
photochlorophyllide reductase subunit chlN	1	3 (1:2:0)	
photosystem II subunit V	2	5 (1:3:1)	2axt(E)
photosystem II subunit VI	2	7 (1:5:1)	2axt(F)
photosystem II CP47 protein	2	55 (15:34:6)	2axt(B)
photosystem II H protein	1	4 (0:4:0)	2axt(H)
photosystem II J protein	1	2 (1:1:0)	2axt(J)
photosystem II L protein	4	8 (1:7:0)	2axt(L)
photosystem II M protein	1	1 (0:1:0)	2axt(M)
photosystem II N protein	1	2 (0:2:0)	
photosystem II T protein	2	5 (2:3:0)	2axt(T)
photosystem II Z protein	1	1 (0:0:1)	2axt(Z)
cytochrome *b*6*f *complex petG	1	1 (0:1:0)	1vf5(G)
cytochrome *b*6*f *complex petL	1	2 (1:1:0)	1vf5(E)
cytochrome *b*6*f *subunit 4	2	19 (4:15:0)	1vf5(B)
cytochrome *f*	1	2 (2:0:0)	1vf5(C)
photosystem I P700 apoprotein A1	1	1 (1:0:0)	1jb0(A)
photosystem I P700 apoprotein A2	1	1 (0:1:0)	1jb0(B)
photosystem I subunit IX	1	1 (0:1:0)	1jb0(J)
photosystem I assembly protein Ycf3	2	9 (2:7:0)	
ATPase α	21	100 (66:28:6)	2ck3(A)
ATPase β	1	7 (1:6:0)	2ck3(D)
ATPase ε	1	3 (1:2:0)	1fs0(E)
ATP synthase CF0 A chain	1	11 (2:8:1)	
ATP synthase CF0 B chain	1	1 (0:1:0)	
ATP synthase CF0 C chain	1	7 (1:6:0)	
ATP synthase subunit 6	12	38 (19:17:2)	
ATP synthase subunit 9	10	58 (17:37:4)	
RUBISCO large subunit	5	35 (11:23:1)	1uw9(A)

**Other machinery**			

Chloroplast envelope membrane protein	1	5 (3:2:0)	
Acetyl-coA carboxylase carboxyl transferase β	1	16 (6:9:1)	2f9i(B)
succinate dehydrogenase subunit 4	1	1 (0:1:0)	
maturase K (intron-encoded protein)	1	4 (0:4:0)	
maturase R (intron-encoded protein)	5	54 (13:36:5)	

**Hypothetical organelle proteins**			

hypothetical protein ycf1	1	2 (0:1:1)	
hypothetical protein ycf2	1	7 (4:2:1)	
hypothetical protein ymf19	5	20 (4:8:8)	
orf114	1	2 (0:0:2)	
orf240a	1	1 (1:0:0)	
orf25	5	45 (8:33:4)	
orfX	4	101 (45:45:11)	

**Total**	365	3560 1219 1983 358	

Detail of the data is described in Additional files 1 and 2.

Classification of the 365 gene products by sequence identity results in 88 protein families (Additional files 1 and 2). There are 1,923 unique RNA editing events; most of these are C-U conversions (Table 2). In Table 2, there are 13 events involving other types of conversions; all of these events were observed in the mRNA encoding mitochondrial cytochrome *b*_6 _from *Pfiesterra piscicida *[22].

**Table 2:** Conversion patterns of nucleotides by RNA editing

	**mRNA**			
**gene**	**A**	**U**	**G**	**C**

**A**	0	0	9	0
**U**	0	0	0	151
**G**	1	0	0	3
**C**	0	1760	0	0

**Figure 1:**
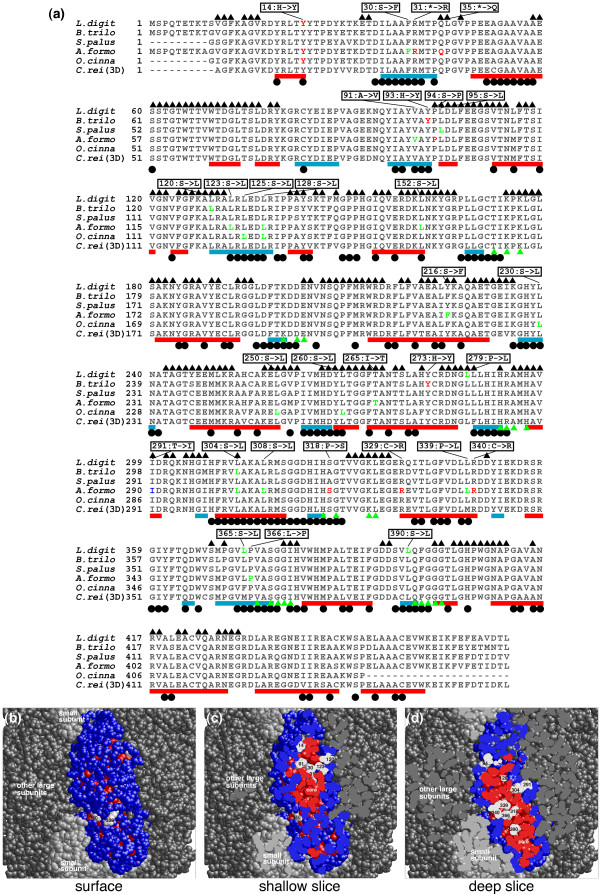
Amino acid residues converted by RNA editing in the RuBisCO large subunit. **(A) **Multiple sequence alignment of RuBisCO large subunit derived from chloroplast genes of *Lycopodium digitatum *(*L. digit*), *Bazzania trilobata *(*B. trilo*), *Sphagnum palustre *(*S. palus*), *Anthoceros formosae *(*A. formo*), *Osmunda cinnamomea *var. *fokiensis *(*O. cinna*) and *Chlamydomonas reinhardtii *(*C. rei*(3D)). Data regarding RNA editing sites were gathered from [10]. Amino acid sequence of *C. reinhardtii *RuBisCO was aligned to the other sequences to assign 3D positions of residues given in Protein Data Bank (ID: 1UW9[26]). Amino acid residues converted by RNA editing are colored as follows: Red indicates that the first nucleotide of the codon is edited, green the second, and cyan the second and the third. A red box below each alignment row indicates a residue in a helix structure; a blue box indicates a residue in a strand structure. A black dot below the row indicates a residue in a structural core; a triangle over the row indicates a residue in the interface for the small subunits or the other large subunits. A green triangle indicates a binding site of an intermediate analogue (2-carboxyarabinitol-1,5- diphosphate). Conversion pattern of amino acid residue by RNA editing is described in the box. **(B) **Three-dimensional structure of RuBisCO large subunit in a supramolecule form. Colored molecule in the center is the large subunit in focus, light grey molecules are RuBisCO small subunits, and deep grey molecules are RuBisCO large subunits. On the molecule in the center, residues in red form the structural core, and residues in white are ones converted by RNA editing. Numbers on white residue correspond to the numbers in (A). **(C) **A cross-section of (B) to depict the structural cores. The slice plane is parallel to the figure page. **(D) **A cross-section of (B). The slice plane is parallel to the figure page and deeper than (C).

These data show that ~90% (= (1219 + 1983)/3560) of RNA editing events are observed on the first or the second nucleotide of a codon; this observation suggests that RNA editing events often change the identity of the encoded amino acid. Table 3 shows the patterns of conversion of amino acid residues in the 1,923 RNA editing events. The top five patterns are Ser-Leu conversion (333) followed by Pro-Leu (325), Ser-Phe (248), Pro-Ser (101) and Arg-Trp (83). These conversions mostly restore evolutionarily conserved amino acid residues found in the multiple sequence alignment of homologous proteins (Fig. 1A). The patterns of conversion shown in Tables 2 and 3 are similar to the ones reported in previous studies [16,23,24].

**Table 3:** Conversion patterns of amino acid residues by RNA editing

	**mRNA**																					
**gene**	**G**	**A**	**P**	**S**	**T**	**I**	**V**	**L**	**C**	**M**	**Y**	**F**	**W**	**H**	**K**	**R**	**E**	**D**	**Q**	**N**	*	**gene**

**G**	3	2		1																		**G**
**A**		12					33															**A**
**P**			15	101				325				52										**P**
**S**			3	28				333				248										**S**
**T**		1			18	36				74												**T**
**I**					4	43	4			1												**I**
**V**		6					22															**V**
**L**			7	15				47				60										**L**
**C**									7							5						**C**
**M**					3																	**M**
**Y**									1		11			5								**Y**
**F**				5				8				51										**F**
**W**				1									1			3						**W**
**H**											72			4								**H**
**K**																						**K**
**R**									70				83			3					7	**R**
**E**																						**E**
**D**																		5				**D**
**Q**																					11	**Q**
**N**				1																2		**N**
*																23			47			*

**gene**	**G**	**A**	**P**	**S**	**T**	**I**	**V**	**L**	**C**	**M**	**Y**	**F**	**W**	**H**	**K**	**R**	**E**	**D**	**Q**	**N**	*	**gene**
		
	**mRNA**																					

A stop codon is depicted by *, and a blank element in the matrix means not observed.

### Protein 3D structures of edited transcripts

Out of the 88 protein families whose mRNAs undergo conversion editing, 52 families contain members for which 3D structure data have been deposited in Protein Data Bank [25]; hence, their 3D structures can be modeled (Table 1). Out of the 1,923 RNA editing sites described above, 755 sites encode residues present in these 3D structures.

### Correlation between functional residues and RNA editing sites

**Figure 2:**
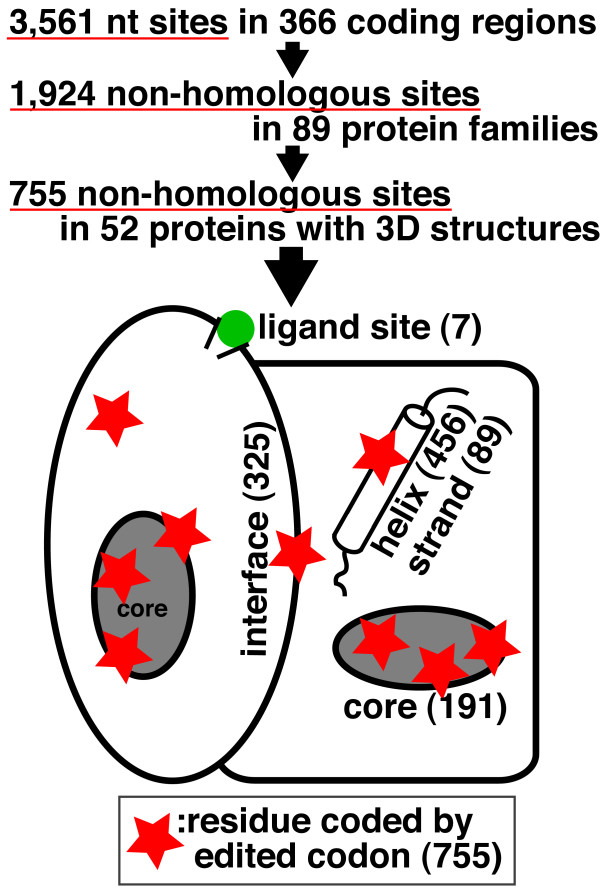
Summary of the relationship between function of the residues and residues encoded by codons with RNA editing. The number in parentheses is the count in 52 protein families.

We have assigned functional residues based on the 3D structures of the 52 proteins, and collated lists of both the functional residues and those residues encoded by edited codons. The correspondence between the edited sites and functional sites is summarized in Figure 2.

#### Ligand-binding residues

We compared the ligand-binding residues and the residues converted by RNA editing in RuBisCO, for which binding sites for a ligand analogue are known [26], and found that none of the residues at the ligand-binding sites are converted by RNA editing (Fig. 1A). Out of the 755 RNA editing sites, only seven sites were found in ligand-binding sites (Additional file 1). First, a Zn-ligand residue in acetyl-CoA carboxylase carboxyltransferase β is encoded by an edited codon. In acetyl-CoA carboxylase carboxyltransferase β encoded by the chloroplast *accD *gene of *Adiantum capillus-veneris*, a CGC codon encoding Arg73 is converted to UGC encoding Cys. The Cys is a part of a zinc finger motif, and the deletion of the motif in plastid *accD *abrogated enzymatic activity [27]. Second, Cu-ligand residues in cytochrome *c *oxidases I and II are encoded by codons undergoing RNA editing. In the mitochondrial *coxI *gene of *Picea abies, Larix *sp., *Megaceros *sp. and *Zamia *sp., a CAU codon encoding His, is converted to UAU encoding Tyr; this Tyr residue bridges an electron transfer pathway from heme to Cu. Replacing Tyr to Phe relocated the Cu, suggesting a role for Tyr in positioning the Cu into the proper electron pathway [28,29]. In many mitochondrial *coxII *genes, an ACG encoding Thr is edited to AUG encoding Met; the Met residue is a ligand for Cu. The Cu ion in *coxII *is also on an electron transfer pathway. When this residue is mutated to Thr, CoxII almost completely loses its ability to bind copper [30]. Third, Fe-S ligand residue in NADH dehydrogenase subunit I is targeted by RNA editing. A CGC codon encoding Arg70 is converted to a UGC encoding Cys in *ndhI *mRNA of chloroplast *A. capillus-veneris*. A Fe-S cluster binding to the Cys residue is a part of an electron transfer pathway [31]. Finally, Mg-ATP binding residue in F1 ATPase α is encoded by an edited codon. A CCU encoding Pro in the mitochondrial gene of *Nicotiana tabacum *is converted to a UCU encoding Ser. However, the residue encodes Pro in the sequence whose 3D structure was determined [32], hence the editing seems to have no effect on the binding of ATP.

#### Residues in secondary structures

Many residues whose codons are converted by RNA editing are found to be a part of secondary structures in proteins, especially helices (Additional file 1). Out of the 755 RNA editing sites, 456 (about 60%) sites are found to locate in an α- or 3_10_-helix structure and 89 locate in a β-sheet structure. The skewed distribution of RNA editing sites on helix is statistically significant (*p *< 8.38 × 10^-9^). In the current data, there are 131 cases in which the residue converted by RNA editing is in a helix but is originally encoded as Pro in the DNA. In these cases, a non-edited amino acid sequence would have a kinked or truncated helix, which would cause a defect in the protein 3D structure (because Pro is a helix-breaker [33]) and presumably result in a less stable protein. Thus, editing repairs plausible defects of the proteins by restoring residues to ones that are appropriate for secondary structure formation.

#### Residues in subunit interfaces

All of the proteins to which we assigned 3D structures in the current study are multi-subunit complexes; stable subunit interactions are an important factor for these proteins to function. RuBisCO consists of an octamer of heterodimers (large and small subunits) with an overall barrel structure. Each large subunit has interfaces to two small subunits and two other large subunits. Residues in the interfaces are shown in Fig. 1A by black triangles. Out of 30 unique residues encoded by codons converted by RNA editing, 13 residues are in the interfaces, and most of them are converted from hydrophilic to hydrophobic residues (Fig. 1). Out of the 755 RNA editing sites in the 52 protein families, 325 sites are located in the interfaces of subunits (Additional file 1). Conversion of Ser to Leu, Ser to Phe and Arg to Trp are respectively found 64, 57 and 23 times in the subunit interfaces. These conversions all switch the physicochemical properties of residues from hydrophilic to hydrophobic. This type of conversion increases the hydrophobicity of interface residues, one of the important properties of protein-protein interfaces [34].

#### Protein structural core

A key step in constructing the 3D structure of a protein is the formation of the structural core. RuBisCO large subunit has 139 (~30%) residues in structural cores (Fig. 1A), and residues converted by RNA editing are biased toward core-forming residues. In Figs. 1B–D, residues converted by RNA editing are shown in white, and those white residues are clustered inside the core-forming residues in red. Out of the 30 unique RNA editing sites in RuBisCO, 18 residues (60%) are located in structural cores, and these sites are over-represented in protein structural cores, considering that only 30% of all residues are in structural cores. The residue of *Anthoceros formosae *RuBisCO corresponding to residue 339 of *Chlamydomonas reinhardtii *RuBisCO (3D known) is originally encoded as Pro, and RNA editing of the codon converts the encoded residue to Leu (Fig. 1A). The residue is a part of a structural core and is located on an α helix. If the residue were not converted by RNA editing, then Pro would disrupt the α helix and the structural core, altering the local volume of the protein even if the resulting peptide was able to fold (Fig. 1D).

Out of the 755 RNA editing sites in the 52 proteins, 648 sites of RNA editing resulted in residue conversion. The other 107 (= 755-648) RNA editing sites are either involved in a stop codon or RNA editing that did not convert amino acid residues. Of the 648 RNA editing sites where the residue is converted, 191 (~30%) are targeted to residues in a protein structural core. In the 52 proteins with 3D structures, there are 12,370 residues and 2,331 residues (about 19%) are included in protein structural cores. Thus, the probability of obtaining the observed distribution is less than 4.54 × 10^-10^. However, since residues in a protein structural core tend to be hydrophobic and RNA editing often converts a residue to a hydrophobic one (Table 3), it could be a natural consequence that the RNA editing sites tend to be located in a protein structural core. We therefore performed the significance test only on Leu and Phe residues of the 52 proteins on all the sequences, and found that the probability is 7.81 × 10^-5^, still significant enough to support the hypothesis that the RNA editing sites are selectively located in codons for residues located within protein structural cores.

## Discussion

We have gathered the known RNA editing sites within protein-coding regions from the nucleotide sequence databases, and compared the location of RNA editing sites to functionally and structurally important sites within these proteins. In previous studies, residues converted by RNA editing were thought to be distributed without any rules in amino acid sequences [15], and only a few cases of RNA editing had been found to target active sites of proteins [16]. In this study, we demonstrate that residues encoded by edited codons are biased toward helices, protein-protein interfaces and protein structural cores. The skew toward helices and protein structural cores are particularly statistically significant. The distributions suggest that the RNA editing sites are located on helices and protein core for some biological reason.

The recent study by Mulligan et al. [35] demonstrated with a sophisticated statistical method that edited codons are frequently grouped within nucleotide sequences, and these groups are separated by long gaps that contain no editing sites. Our analyses here may give structural context to their non-random distribution of edited sites. The groups of edited codons along the amino acid sequence might correspond to a cluster of residues within the protein structural core, and the long gaps between the groups of edited codons may correspond to the region that do not form a protein structural core, i.e., surfaces.

### Functional impact of RNA editing through protein 3D structures

Mutation in a helix is known to have significant impact on protein stability. In T4 lysozyme, introduction of Pro into a helix resulted in significant kink structure and decreased stability by approximately 2.5 kcal/mol [36]. Considering that the overall thermodynamic stability of protein is achieved by a free energy difference of ~5–15 kcal/mol[37], such a decrease in free energy difference between folded and unfolded states of the peptide could easily have an impact on the stability of a protein. An artificial back-mutation of Leu to Pro in the RNA editing site in a helix of cytochrome *b*_6 _was carried out; the authors of that study found that the mutated protein could not form a protein complex, apparently because cytochrome *b*_6 _itself did not fold appropriately [20]. The direct translation of the original DNA sequences, therefore, seems to have repercussion in biological function through protein stability and/or quaternary structure formation; RNA editing restores the residues to make the protein stable and functional.

A mutation in a protein structural core can also have significant impact on protein stability. A mutation of Leu to Ser in a core, one of the typical conversions repaired by RNA editing, is equivalent to the loss of one methyl group and introduction of one hydroxyl group in a side chain of the residue; hence formation of hydrophilic cavity in a protein structural core is expected. The effect of cavity formation in a protein has been experimentally measured; it decreases stability ~3.3 kcal/mol [38]. Alteration of Val to Ser in the core of ribonuclease T1 decreased stability of the protein by 4.7 kcal/mol [39]. Two to three cavities in a protein structural core, expected in the unedited polypeptide, may therefore have a significant effect on protein stability. Sakaki et al. [21] expressed both edited and unedited acetyl-coA carboxylase carboxyl transferases β of pea, which had Leu and Ser, respectively, at residue 267, and measured the function of the protein in a complex with the α subunit. In addition to detecting no activity in the unedited complex, the authors found that the solubility of the unedited complex was low compared to the edited complex. Freezing and thawing of the eluate affected the unedited complex and resulted in an insoluble complex, whereas the disturbance did not affect the edited complex. Based on our 3D structure analysis, residue 267 is in a protein structural core; hence, this experiment directly showed that RNA editing in a structural core governs the stability of a protein. In the mitochondrion of *Z. mays*, the unedited mRNA encoding ribosomal protein S12 is translated into a polypeptide that cannot be incorporated into ribosomes [40]. The product from the unedited mRNA had hydrophilic residues in place of hydrophobic residues required for protein core formation, and therefore would not form a structure stable enough to participate in supramolecule formation. Islas-Osuna et al. [41] suggested that several of the RNA editing sites on cytochrome *b *from grapevine were located either in the protein structural core or the interfaces with other subunits. Their analysis of one specific protein is consistent with our whole data analyses.

The conversion of amino acid residues within helices and structural cores by RNA editing restores residues that contribute to the formation of stable 3D structure. The unedited products are generally unstable or do not fold, and could disturb the protein networks in organelles that the proteins involve. A mechanism to post-transcriptionally switch amino acids within a protein core could be used to regulate functionality of the protein without regulating transcription of the gene for the protein. In addition, a mixture of edited and unedited products in a cell could yield multiple proteins with different stabilities, all derived from a single gene. If the ratio of edited products in the cell can be modified by regulation of RNA editing enzymes encoded in the nuclear genome [42,43], then the efficiency of the biological functions involving the edited products can be controlled post-transcriptionally.

### Implications for the evolution of RNA editing

The similarities between conversion editing observed in mitochondrion and chloroplast suggests that both mechanisms originated from a common source [16]. Combining the discussions in the previous studies summarized below, we propose an evolutionary scenario for the origins of RNA editing in plant organelles that differs from the previously proposed ones [44,45].

Three sets of observations may bear on discussions of the evolution of the mechanism of RNA editing in organelles. First, it has long been noted that the target C of RNA editing is often followed by a pyrimidine nucleotide, usually U [16]. We counted the number of each nucleotide before and after the edited C on DNA in our dataset, and found that at the preceding site, T far exceeds the other types of nucleotides (Table 4). This bias of T suggests that the RNA editing recovers TT dinucleotide sequences that are underrepresented on DNA. Second, genome sequences of plant mitochondria are known to be GC rich, and there is a positive correlation between the number of RNA editing sites in mitochondrial genomes and their G+C content [46]. RNA editing was then suggested to be a mechanism to compensate for the genetic drift from T to C [46], namely to counteract for GC pressure. The cause of GC pressure may vary, but one of the suggested physical causes is to avoid pyrimidine dimmer formation [47]. Third, RNA editing in organelles has been almost exclusively found in land plants. Yoshinaga et al. proposed that RNA editing in chloroplasts had been acquired to effect the land adaptation of plants [11]. When plants started to migrate to the land (about 0.4 billion years ago), the land was yet to be protected against ultraviolet (UV) light by the ozone layer [48,49]. Whereas aquatic plants are protected by water from the hazardous effects of UV light (i.e., the formation of pyrimidine dimers on DNA) [50], land plants required a novel means to protect themselves.

**Table 4:** Frequency of the preceding and following nucleotide types at C-U conversion sites

	*i*-1	*i*	*i*+1	
A	167	C	526	A
T	1079		429	T
G	64		583	G
C	574		346	C

The studies cited above conjectured that introduction of RNA editing in plant organelle was a positively selected countermeasure against pyrimidine dimer formation. In that case, RNA editing should have been introduced at almost all TT sites. After the reduction of UV light by ozone layer formation, RNA editing sites at the protein cores remained, while RNA editing sites that were not essential to protein structure formation, namely ones located on the surface, could disappear in a neutral manner. If direct translation of the encoded residue has little effect on protein 3D structure stability, then RNA editing on the site could disappear. The correlation between residues converted by RNA editing and their positions in protein structures could have emerged as an outcome of the process.

A different scenario for emergence of RNA editing can be considered. Mitochondria and chloroplasts are the primary sites for oxygen burning and oxygen production, respectively. Molecular oxygen can causes alterations in the chemical structure of the bases in DNA. In order to mitigate the effect of oxygen molecules on organellar DNAs, it would have been effective to both reduce the number of DNAs in the organellar genomes by gene transfer to the nucleus, and to compensate for mutations within organellar DNAs by RNA editing. In animal mitochondria, gene transfer to the nuclear genome has been observed. In plant organelles, both gene transfer and alteration of T to C on DNAs have been observed. T to C conversion on DNA would reduce the number of AT base pairs, which are more susceptible to oxygen molecules than GC base pairs [51].

## Conclusion

We found statistically significant correlations between residues encoded by edited codons and the residues responsible for secondary structure and protein structural core formation. The correlation suggests that RNA editing affects protein functions indirectly by regulating protein stability as well as sometimes being essential for protein enzymatic activity. By repairing the nucleotide sequence of mRNA to encode a stable protein, the RNA editing machinery may regulate expression of protein functions in plant organelles.

## Methods

### Collection of data for conversion RNA editing

The descriptions of RNA editing sites in Genbank/EMBL/DDBJ [52-54] are not standardized, and there are a number of efforts (including the one here) to launch a database for RNA editing sites [55,56]. In order to identify RNA editing sites, we performed a full-text search to find a string of characters that matches both "RNA" and "editing" in "/note" of "misc_feature" line of Genbank database release 158. We wrote a computer program to extract entries with RNA editing in protein-coding regions, and translated both edited and unedited mRNAs into amino acid sequences. We have encountered a significant number of errors in the Genbank annotation during this process, and corrected these annotations based on either literature or communication with depositors. Most of the errors took the form of discrepancies between the nucleotide position number described in misc_feature line and the RNA editing sites in deposited nucleotide sequences. The remediation was carried out on AB254134, AJ006146, AY820131, AY521591, BA000029, DQ645537, DQ984517, X69720, X92735 and Y17812. We could not correct all the errors we encountered, because we could not contact all the depositors. The entries with apparent errors were discarded. For RNA editing on *rbcL *transcripts, we copied the RNA editing site description into the following entries from a table found in reference [10]; D14882, D43696, L11055, L11056, and L13485. When we encountered a pair of protein sequences with identical amino acid sequences and identical RNA editing patterns in their mRNAs, we eliminated one of the entries from our dataset.

### Protein 3D structures of RNA edited transcripts

We performed a homology search of amino acid sequences predicted from edited mRNAs against amino acid sequences of the protein with known 3D structures in PDB [25] using BLAST [57]. When the sequence identity was 25% or more, we used the 3D structures in PDB for assigning structural properties of the products encoded by the edited and unedited mRNAs.

### Functional residues on proteins

We gathered four types of protein functions based on the literature and protein 3D structures. (1) Ligand-binding residues: Most of the protein 3D structures were determined with their ligands and cofactors. Residues that bind those ligands were determined based on measuring solvent accessibilities of a residue with and without the ligand. When the difference between solvent accessibility of a residue calculated with and without the ligand was non-vanishing, then the residue was assigned as a ligand-binding residue. Solvent accessibility was calculated using a modified method of Sharke and Rupley [58] with water radius of 1.4Å. (2) Protein-protein interfaces: Most of the protein products of edited mRNAs are components of supramolecules, and their 3D structures were determined in protein complex form. Residues that interact with other subunits were determined by measuring the solvent accessibility of a residue with and without other subunits. (3) Secondary structure: We assigned secondary structures using DSSP [59]. (4) Protein structural core: A structural core was determined using the following procedure; i) Calculate solvent accessibility, find residues with zero accessibility, and calculate all of the carbon atom distances between these residues; if the distance is no more than 4.0Å, then the pair of residues are parts of the protein structural core. ii) Calculate solvent accessibility, find residues with accessibility more than zero but no more than 0.05, calculate the distance between a carbon atom in the residue and a carbon atom in i); if the distances are 4.0Å or less, then the residue is a part of the protein structural core.

## Authors' contributions

KY built the database, calculated the correlation and drafted the manuscript. MG started and supervised the study. All authors read and approved the final manuscript.

## Supplementary Material

Additional file 1 **supplementary table 1**. A list of residues converted by RNA editing in 52 proteins with known 3D structures. The table also contains secondary structures, solvent accessibility and the species name from which the gene was derived. Click here for file

Additional file 2 **supplementary table 2**. A list of residues converted by RNA editing in 37 proteins without known 3D structures. Click here for file
